# Influence of dental status on postoperative complications in major visceral surgical and organ transplantation procedures—the bellydent retrospective observational study

**DOI:** 10.1007/s00423-024-03448-z

**Published:** 2024-09-19

**Authors:** Anastasia Spitzner, Markus Mieth, Ewan A. Langan, Markus W. Büchler, Christoph Michalski, Franck Billmann

**Affiliations:** 1 Praxis Dr. Dietmar Czech, Marktplatz 15, 16, 89073 Ulm, Germany; 2 Department of General, Visceral and Transplantation Surgery, University Hospital Heidelberg, Im Neuenheimer Feld 420, 69120 Heidelberg, Germany; 3 Department of Dermatology and Venerology, University Hospital Schleswig Holstein, Campus Lübeck, Ratzeburger Allee 160, 23538 Lübeck, Germany; 4 Botton-Champalimaud Pancreatic Cancer Center, Champalimaud Foundation, Avenida Brasília, 1400–038 Lisboa, Portugal; 5 Dermatological Sciences, University of Manchester, Manchester, UK

**Keywords:** Oral health, Oral hygiene, Morbidity, Surgery, Infectious complications, Transplantation, Dental status

## Abstract

**Purpose:**

The significance of dental status and oral hygiene on a range of medical conditions is well-recognised. However, the correlation between periodontitis, oral bacterial dysbiosis and visceral surgical outcomes is less well established. To this end, we study sought to determine the influence of dental health and oral hygiene on the rates of postoperative complications following major visceral and transplant surgery in an exploratory, single-center, retrospective, non-interventional study.

**Methods:**

Our retrospective non-interventional study was conducted at the Department of General, Visceral, and Transplant Surgery, University Hospital Heidelberg, Germany. Patients operated on between January 2018 and December 2019 were retrospectively enrolled in the study based on inclusion (minimum age of 18 years, surgery at our Department, intensive care / IMC treatment after major surgery, availability of patient-specific preoperative dental status assessment, documentation of postoperative complications) and exclusion criteria (minor patients or legally incapacitated patients, lack of intensive care or intermediate care (IMC) monitoring, incomplete documentation of preoperative dental status, intestinal surgery with potential intraoperative contamination of the site by intestinal microbes, pre-existing preoperative infection, absence of data regarding the primary endpoints of the study). The primary study endpoint was the incidence of postoperative complications. Secondary study endpoints were: 30-day mortality, length of hospital stay, duration of intensive care stay, Incidence of infectious complications, the microbial spectrum of infectious complication. A bacteriology examination was added whenever possible (if and only if the examination was safe for the patient)for infectious complications.

**Results:**

The final patient cohort consisted of 417 patients. While dental status did not show an influence (*p* = 0.73) on postoperative complications, BMI (*p* = 0.035), age (*p* = 0.049) and quick (*p* = 0.033) were shown to be significant prognostic factors. There was significant association between oral health and the rate of infectious complications for all surgical procedures (*p* = 0.034), excluding transplant surgery. However, this did not result in increased 30-day mortality rates, prolonged intensive care unit treatment or an increase in the length of hospital stay (LOS) for the cohort as a whole. In contrast there was a significant correlation between the presence of oral pathogens and postoperative complications for a group as a whole (*p* < 0.001) and the visceral surgery subgroup (*p* < 0.001). Whilst this was not the case in the cohort who underwent transplant surgery, there was a correlation between oral health and LOS in this subgroup (*p* = 0.040). Bacterial swabs supports the link between poor oral health and infectious morbidity.

**Conclusions:**

Dental status was a significant predictor of postoperative infectious complications in this visceral surgery cohort. This study highlights the importance preoperative dental assessment and treatment prior to major surgery, particularly in the case of elective surgical procedures. Further research is required to determine the effect of oral health on surgical outcomes in order to inform future practice.

**Trial registration:**

Trial registered under the ethics-number S-082/2022 (Ethic Committee of the University Heidelberg).

## Introduction

The link between oral health and hygiene and several systemic inflammatory disease, including psoriasis, is becoming more widely appreciated. However, the influence of oral health on surgical outcomes is less well recognized. In fact, there is clear evidence that oral hygiene and oral microbial dysbiosis impacts signficnantly on surgical outcome measures [1, 2]. For example, poor pre- and perioperative oral health and infection can lead to several postoperative complications [2–11] including, impaired wound healing, increased rates of wound infection and development of cardiovascular and cerebrovascular complications including endocarditis and septic emboli respectively [10, 12]. In turn, such complications can translate into prolgonged periods of hospitalization (increased length of hospital stay (LOS)), with the incumbent increase in treatment costs. Moreover prolonged hospitalization impacts negatively on quality of life with increased postoperative patient morbidity and mortality [1, 2, 8, 9, 11].

According to the latest World Health Organisation "Global oral health status report," almost 50% of the population suffers from untreated dieases of the oral cavity [13–15]. The oral microbial biofilm plays a crucial role in this regard [16, 17]. Poor oral hygiene can lead to changes in the composition of the oral microbiome (dysbiosis) and may trigger subclinical inflammation or promote acute infection [18, 19]. A range of common medical conditions have been assosicated with poor oral health and hygiene, including diabetes [20], dementia [21] and cerebro- and cardiovascular disorder [22–24] conditions. Therefore, whilst poor oral hygiene must result from a range of medical conditions, it may itself trigger and/or exacerbate systemic inflammatory disease and result in systemic infection.

This association is of particular importance in the surgical context, where poor oral health appears to increase the risk of postoperative sepsis [25] and pneumonia [4]. More specifically, oral disease is linked to graft infection in vascular surgery [5], prosthetic infection in orthopedic surgery [3, 5, 10, 26], surgical site infections (SSI) and pneumonia after major gastrointestinal and thoraco-abdominal surgeries [6, 7, 9, 11] and endocarditis following cardiothoracic  surgery for heart valve replacement or reconstruction [27, 28]. In contrast, preoperative treatment of oral disease has been shown to prevent postoperative complications in both prospective studies and meta-analyses^8^.

However, the data on this association between oral health and post surgical complications remains scare, often based on small case-series, and subject to significant bias as a results of the methodologies which were employed [1, 6–9, 29, 30].

Therefore, we sought to further delineate the association between preoperative oral health and postoperative complications in a large cohort of oncological and non-oncological surgical patients and patients undergoing organ transplantation surgery in order to determine whether complication rates were surgical procedure-specific.

## Materials and methods

### Study design

This is a single-center, retrospective, exploratory, non-interventional study conducted at the Department of General, Visceral, and Transplant Surgery, University Hospital Heidelberg, Germany. All patients were treated according to standard established medical and surgical practices. No study-specific interventions were performed. Our study was registered and was approved by the Ethic Committee of the University of Heidelberg (Ethics-Number S-082/2022).

### Patient cohort, inclusion and exclusion criteria

The patient cohort consisted of 427 patients. Inclusion criteria were: (1) Minimum age of 18 years, (2) surgery at the Department of General, Visceral, and Transplant Surgery, University of Heidelberg, between January 2018 and December 2019, (3) Intensive care / IMC treatment after major surgery at University Hospital Heidelberg. This criterion was established for patient inclusion to ensure reliable documentation of dental status, postoperative complications, and bacteriological/microbiological data. Major surgery was defined as any procedure in an operating room requiring the use of general anesthesia for a nonpercutaneous nonendoscopic invasive operation as previously published [31, 32], (4) Availability of patient-specific preoperative dental status assessment (anesthesia record, preoperative dental examination), (5) Documentation of postoperative complications, particularly infectious complications with corresponding microbioligcal investigations (bacteriological examination with antibiogram). Exclusion criteria were: (1) Minor patients or legally incapacitated patients, (2) lack of intensive care or intermediate care (IMC) monitoring, (3) incomplete documentation of preoperative dental status, (4) intestinal surgery with potential intraoperative contamination of the site by intestinal microbes (e.g., rectal resection, sigmoid resection), (5) pre-existing preoperative infection (e.g., abscess, pneumonia, sepsis), (6) absence of data regarding the primary endpoint of the study.

### Primary and secondary study endpoints

The study-relevant endpoints were retrospectively collected from the university's clinical information system (I.S.H. med Clinical System, SAP SE Walldorf, Dietmar-Hopp-Allee 16, 69190 Walldorf).

The primary study endpoint was the incidence of postoperative complications (postoperative morbidity). These were early complications occurring within the first 30 days after surgery, categorized according to the Clavien-Dindo classification [33]. Infectious complications were identified in both the discharge letters and following careful review of the daily documentation entered by the responsible clinicians software ISH. med (SAP SE, Walldorf) and COPRA (COPRA System GmbH, Berlin).

The dental status of the operated patients was assessed by the IMC/ICU physician during the initial post-operative standardized examination and classified into the categories “not assessable”, “normal/restored”, “prosthetics”, “loose” and “damaged”. The category “not assessable” was excluded from the study. Patients with a dental status of “normal/restored” were rated as “good dental status”, while the categories of “prosthesis”, “loose” and “damaged” were rated as “poor dental status”.

The secondary study endpoints were defined as follows: (1) 30-day mortality (the absolute number of deaths in the first 30 days following the procedure and the percentage of death relative to the number of procedures performed, (2) duration of hospital stay or length of hospital stay (i.e. the period between date of admission and date of discharge), (3) duration of intensive care stay, (4) Incidence of infectious complications, (5) the microbial spectrum of infectious complication. In addition to the secondary endpoints, patient-specific demographic data and surgery-specific data were collected: (1) age (2) sex; (3) body mass index; (4) comorbidities according to the Charlson comorbidity index [34]; (5) preoperative medication (particularly immunosuppressive medication including corticosteroids (6) preoperative laboratory parameters (coagulation, liver values, leucocytes, inflammatory parameters), (7) the presence of ascites. Pre- and post-operative clinical chemistry variables (especially liver, renal, coagulation, leucocytes and inflammation parameters) were evaluated using the laboratory module of the hospital medical software. The following parameters were evaluated: (1) GOT (U/L), (2) GPT (U/L), (3) AP (U/L) (4) Bilirubin (mg/dL), (5) Leucocytes ( /nL), (6) CRP (mg/dL), (7) Quick (%).

Operation-specific data were collected: (1) type and extent of operation, (2) duration of the operation; (3) administration of preoperative antimicrobial prophylaxis, (4) estimated blood loss.

### Data management

Patient data were collected and managed in a Microsoft Excel 2016 file (Microsoft, Redmond, USA). Data were pseudonymized and stored in an Excel table. Collection and storage complied with the Federal Data Protection Act (LDSG) of 30 June 2017 [35]. Data processing was carried out in accordance with legal provisions. The study was approved by the local ethics committee (ethics number: S-082/2022).

### Statistical analysis

Statistical analysis was performed using SPSS 29.0.0.0 software (IBM, 1 New Orchard Road, Armonk, New York 10504–1722, United States). The analysis of frequency distribution of age, gender, BMI, pre-existing conditions, Charlson Comorbidity Index, microbial data, and postoperative complications was conducted using descriptive statistics. Categorical variables were presented as absolute and relative frequencies, while continuous variables were calculated as means and standard deviations. Logistic regressions were conducted to assess the influence of individual variables on postoperative complications. These regressions were applied as sensitivity analysis both to the overall cohort and to the subgroups of visceral surgery without solid organ transplantation (SOT) and transplantation surgery. Spearman's correlation was used to analyze the relationship between oral microbes and postoperative complications, given the categorical nature of the variables. The statistical significance level was set at *p* < 0.05 for all models. Patients wearing complete or partial dentures were classified as having poor dental status. This classification is based on several studies that followed the same approach [28, 30, 36, 37]. This is grounded on the assumption that an oral disease, such as caries or periodontitis, leads to tooth loss and thus indicates an existing poor dental status. The decision to analyze subgroups is justified by the use of pre-, peri-, and post-operative steroids in transplantation surgery to prevent acute and chronic transplant rejections [38, 39]. However, such immunosuppression impact profoundly on postoperative outcomes, potentially reduction in under-recognition of complications. Furthermore, due to occurrences of endocarditis in cardiac surgery after heart valve replacement or reconstruction, the discussion extends to whether dental status also holds significance in transplantation surgery [9, 27, 28, 40–42].

## Results

### Descriptive analysis

**Fig. 1:**
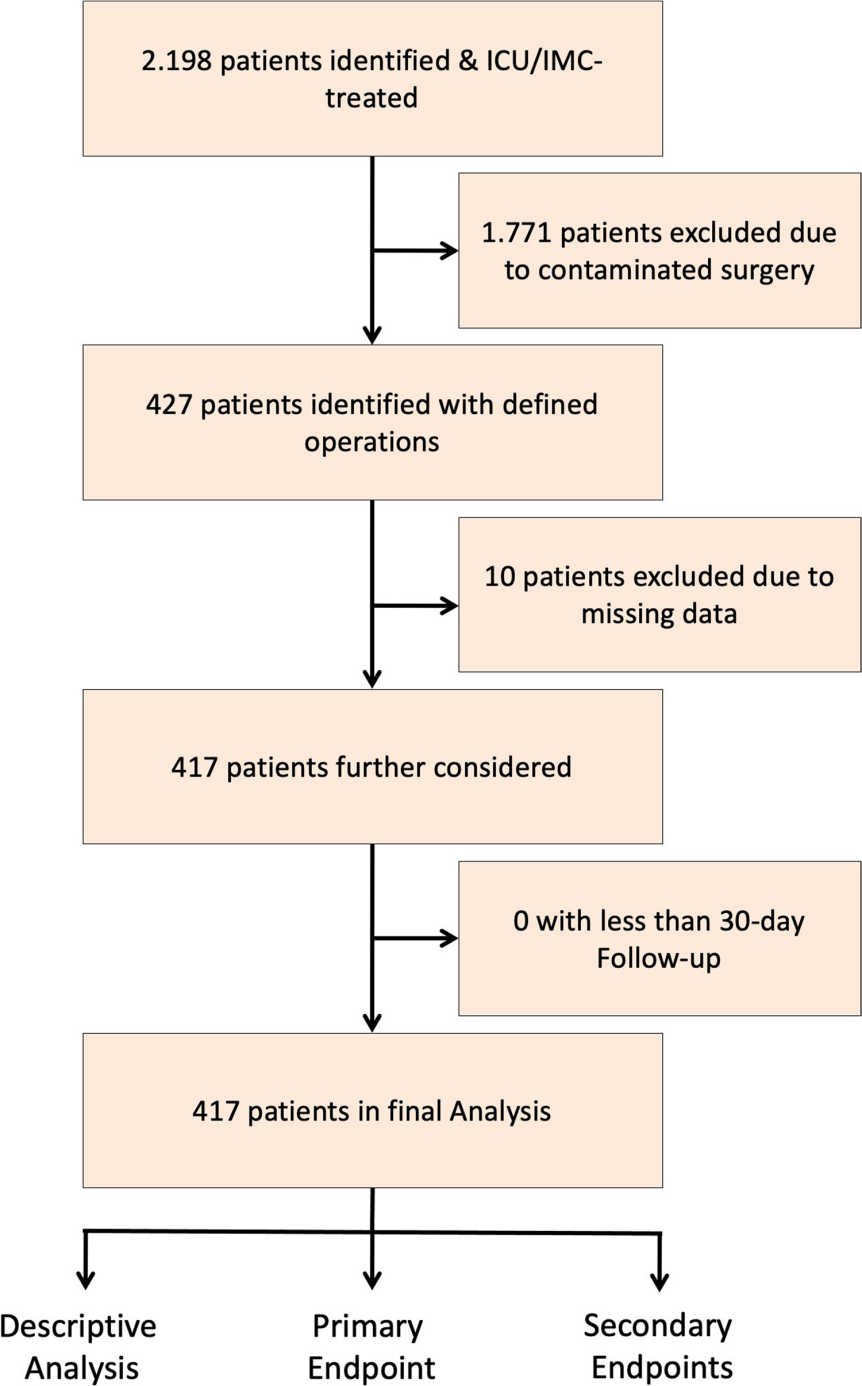
Flowchart of the present study

The initial patient cohort consisted of 427 patients. Ten patients were subsequently excluded due to incomplete data. The study flowchart is presented in Fig. 1. The results of the descriptive analysis are summarized in Table 1. Of the 417 patients analysed, 247 (59.2%) were male and 170 (40.8%) were female. A total of 639 procedures were performed, with one patient potentially undergoing more than one procedure per operation (Table 1). The median age was 59.5 years with a standard deviation of 14.1 years. 17 patients (4.0%) were underweight, 178 patients (42.7%) were normal weight, with 140 patients (33.6%) and 82 (19.7%) respesrtively overweight and obese according to the BMI. 65.7% of patients were classified as CCI category 5 and above, 27.8% CCI category 3–4 and 5.3% were CCI category 1–2. Only 1.2% of patients were classified as CCI category 0. In the transplant surgery cohort, 31 (27.9%) were liver transplants, 73 (65.8%) were kidney transplants and 7 (6.3%) were combined kidney and pancreas transplants. Of these 111 cases (n = 111), 61 were male (55%) and 50 (45%) were female. The dental status was assessed as good in 312 patients (74.8%) and poor in 105 patients (25.2%).

**Table 1:** Descriptive Statistics of the 417 patients included in the final analysis

Variables	*n* (%)
Sex Female/Male	170 (40,8) / 247 (59,2)
Age, yrs	
< 50	90 (21,6)
50—60	97 (23,3)
60—70	118 (28,3)
70—80	98 (23,5)
> 80	14 (3,3)
BMI, kg/m2	
< 18,5	17 (4,0)
18,5—25	178 (42,7)
25—30	140 (33,6)
> 30	82 (19,7)
Charlson Comorbidity Index	
0	5 (1,2)
1—2	22 (5,3)
3—4	116 (27,8)
≥ 5	274 (65,7)
Comorbidities (detail)	
Myocardial infarction	15 (3,6)
Cardial insufficiency	9 (2,2)
Peripheral arterial occlusive disease	16 (3,8)
Transitory Ischemic Attack	14 (3,4)
Dementia	1 (0,2)
COPD	26 (6,2)
Connective tissue disease	8 (1,9)
GERD/gastric ulceration	202 (48,4)
Liver disease	116 (27,8)
Diabetes mellitus	87 (20,9)
Hemiplegia	3 (0,7)
Chronic renal insufficiency	121 (29,0)
Malignant disease (Carcinoma)	288 (69,1)
Lymphoma	1 (0,2)
Operations (detail) (total *n* = 639)	
Adrenalectomy	11 (1,1)
Cholecystectomy	131 (12,5)
Duodenectomy	1 (0,1)
Duodenotomy	2 (0,2)
Fundoplication	3 (0,3)
Gastrectomy	39 (3,7)
Hepatectomy	56 (5,3)
Hernia Operation	5 (0,5)
Lymphadenectomy	3 (0,3)
Nephrectomy	7 (0,7)
Esophagectomy	29 (2,8)
Pancreatectomy	163 (15,6)
Parathyreoidectomy	2 (0,2)
Biopsy	7 (0,7)
Peritonectomy	1 (0,2)
Splenectomy	65 (6,2)
Thyreoidectomy	3 (0.3)
Transplantation	111 (10,6)
Liver	31 (3)
Kidney	73 (7)
Kidney + Pancreas	7 (0,7)

### Primary endpoint: postoperative morbidity

In the entire cohort postoperative complications occurred in a total of 230 (47.5%) patients. A total of 397 complications could be identified (one patient could develop one or more complications). Of these, 32 (8.0%) were gastrointestinal, 46 (11.6%) cardiac, 59 (14.8%) pulmonary and 48 (12.1%) renal complications. An overview of the distribution of these complications is provided in Table 2. According to the Clavien Dindo classification, 6 (1.4%) patients were assigned to class 1, 72 (17.3%) patients to class 2, 27 (6.5%) to Class 3a, 69 (16.5%) to Class 3b, 40 (9.4%) to Class 4a, 6 (1.4%) to Class 4b and 10 (2.3%) patients to Class 5. Dental status did not have a significant influence on the occurrence of postoperative complications (*p* = 0.73). In contrast, age (*p* = 0.049), BMI (*p* = 0.035) and quick value (*p* = 0.033) were significantly related to the incidence of postoperative complication (Table 3). In 121 patients (29%) the complications were infection-related in nature (e. g. surgical site infections—SSIs, sepsis and pneumonia) and accounted for 45.3% of the total complications (n = 180). Logistic regression analysis showed a significant influence of dental status (*p* = 0.034) and duration of surgery (*p* = 0.027) on the occurrence of postoperative infection-related complications. Table 4 provides an overview of this analysis.

**Table 2:** Postoperative Morbidity in the included patient cohort

Complications	*n* (%)*
Ileus	6 (1,5)
Colitis	3 (0,8)
Gastric ischaemia	6 (1,6)
Intestinal transit disorder	17 (4,7)
STEMI/NSTEMI	12 (3,3)
Atrial fibrillation and arrhythmia	24 (6,6)
Heart failure	2 (0,5)
Asystole	3 (0,8)
Haemorragic shock	5 (1,4)
Pneumonia	30 (8,2)
Respiratory insufficiency /ARDS	18 (4,9)
Pleural empyema	1 (0,3)
Pulmonary embolism	2 (0,5)
Atelectasis	3 (0,8)
Pneumothorax	5 (1,4)
Urinary infection	24 (6,6)
Acute renal failure	24 (6,6)
Wound healing disorder	57 (15,6)
Surgical site infection—SSI	102 (27,9)
Sepsis	21 (5,8)
Postoperative haemorrhage	32 (8,1)

* % indicate the percentage related to total complications, not the incidence of the complication in the patient cohort

**Table 3:** Binary logistic Regression – Total morbidity in the included 417 patients

Variables	Regression-coefficient B	Standard-error	Wald	df	Sig	Exp(B)
Age (yrs)	,019	,010	3,884	1	,049	1,019
Sex	-,213	,218	,959	1	,327	,808
BMI (Kg/m2)	,045	,021	4,447	1	,035	1,046
Dental status	,473	,264	3,211	1	,073	1,605
Charlson Comorbidity Index	,041	,052	,637	1	,425	1,042
Preop. steroid medication	-,346	,268	1,669	1	,196	,707
Preop. ascites	,475	,519	,836	1	,361	1,607
Coag. disorder (Quick,%)	-,014	,007	4,528	1	,033	,986
Preop. Bilirubin (mg/dL)	,107	,068	2,494	1	,114	1,113
Preop. GOT (U/L)	,003	,002	1,163	1	,281	1,003
Preeop. GPT (U/L)	-,001	,001	1,158	1	,282	,999
Preop. AP (U/L)	-,001	,001	2,013	1	,156	,999
Preop. Leucocytes (/nL)	,028	,042	,450	1	,502	1,029
Preop. CRP (mg/dL)	-,005	,005	,940	1	,332	,995
Open vs. MIC access	,153	,513	,089	1	,765	1,166
OP-Duration (min)	,001	,001	1,756	1	,185	1,001

**Table 4:** Binary logistic Regression – Infectious complications in the included 417 patients

Variables	Regression-coefficient B	Standard-error	Wald	df	Sig	Exp(B)
Age (yrs)	,002	,011	,055	1	,814	1,003
Sex	-,110	,237	,215	1	,643	,896
BMI (Kg/m2)	,019	,022	,792	1	,374	1,020
Dental status	,555	,262	4,479	1	,034	1,741
Charlson Comorbidity Index	,089	,054	2,419	1	,120	1,087
Preop. steroid medication	-,393	,305	1,661	1	,197	,675
Preop. ascites	,564	,474	1,416	1	,234	1,759
Coag. disorder (Quick,%)	-,002	,007	,126	1	,722	1,002
Preop. Bilirubin (mg/dL)	,045	,031	2,141	1	,143	1,046
Preop. GOT (U/L)	,000	,001	,075	1	,784	1,000
Preeop. GPT (U/L)	,001	,001	1,081	1	,299	1,001
Preop. AP (U/L)	,000	,001	,000	1	1,000	1,000
Preop. Leucocytes (/nL)	-,020	,043	,220	1	,639	,980
Preop. CRP (mg/dL)	,002	,004	,139	1	,710	1,002
OP-Duration (min)	,002	,001	4,883	1	,027	1,002

A total of 345 postoperative complications occurred in 182 (59.5%) patients in the visceral surgery cohort,. Of these, 26 (7.5%) were gastrointestinal, 44 (12.8%) cardiac, 61 (17.7%) pulmonary and 39 (11.3%) renal. In addition, there were 44 (12.8%) cases of wound healing disorders, 89 (25.8%) cases of SSIs, 21 (6.1%) cases of sepsis and 21 (6.1%) cases of postoperative bleeding. This study showed that the BMI (*p* = 0.033) and the preoperative incidence of preoperative ascites value (*p* = 0.035) had a significant influence on the incidence of postoperative complications. 97 (31.7%) patients experienced an infectious complication postoperatively. The dental status (*p* = 0.043) had a significant influence on the occurrence of infectious complications in this cohort (Table 5). So did preoperative ascites (*p* = 0.006), bleeding diatheses (*p* = 0.021) and preoperative elevated blood levels of bilirubin (*p* = 0.008) and GPT (*p* = 0.041).

**Table 5:** Binary logistic Regression – Infectious complications in the visceral surgery cohort

Variables	Regression-coefficient B	Standard-error	Wald	df	Sig	Exp(B)
Age (yrs)	,007	,013	,326	1	,568	1,008
Sex	-,295	,281	1,103	1	,294	,745
BMI (Kg/m2)	,020	,025	,648	1	,421	1,020
Dental status	,603	,298	4,108	1	,043	1,828
Charlson Comorbidity Index	,070	,059	1,392	1	,238	1,072
Preop. steroid medication	,387	,645	,360	1	,549	1,473
Preop. ascites	1,982	,727	7,434	1	,006	7,259
Coag. disorder (Quick,%)	,021	,009	5,305	1	,021	1,021
Preop. Bilirubin (mg/dL)	,224	,085	6,934	1	,008	1,252
Preop. GOT (U/L)	,002	,002	,956	1	,328	1,002
Preeop. GPT (U/L)	-,005	,002	4,157	1	,041	,995
Preop. AP (U/L)	-,001	,001	,298	1	,585	,999
Preop. Leucocytes (/nL)	-,017	,050	,114	1	,736	,983
Preop. CRP (mg/dL)	,005	,005	1,048	1	,306	1,005
OP-Duration (min)	,002	,001	2,903	1	,088	1,002

In the transplant surgery cohort, 48 (43.2%) patients experienced postoperative complications. In the Clavien-Dindo scoring system, 13 patients (27.1%) were in group 2, 4 (8.3%) were in group 3a, 21 (43.8%) were in group 3b, 9 (18.8%) were in group 4a and 1 (2.1%) were in group 4b. A total of 64 complications occurred in these 48 patients: 6 patients (9.4%) developed a gastrointestinal complication, 2 (3.1%) developed a cardiac complication, 11 (17.2%) developed a pulmonary complication, and 8 (12.5%) developed a renal complication. In addition, 12 (18.8%) wound healing disorders, 13 (20.3%) SSIs and 12 (18.8%) follow-up hemorrhages occurred. Preoperative ascites (*p* = 0.008), quick (*p* = 0.020), GOT (*p* = 0.026) and GPT (*p* = 0.034) were identified as influencing variables on postoperative complications. Twenty-four patients (21.6%) had postoperative infectious complications. Only the duration of the operation (*p* = 0.041) had a significant influence on the occurrence of infectious complications (Table 6).

**Table 6:** Binary logistic Regression – Infectious complications in the transplantation surgery cohort

Variables	Regression-coefficient B	Standard-error	Wald	df	Sig	Exp(B)
Age (yrs)	-,004	,029	,022	1	,882	,996
Sex	,259	,616	,176	1	,674	1,295
BMI (Kg/m2)	,004	,070	,003	1	,959	1,004
Dental status	,717	,746	,924	1	,336	2,048
Charlson Comorbidity Index	,037	,233	,026	1	,873	1,038
Preop. steroid medication	-1,481	1,255	1,392	1	,238	,227
Preop. ascites	-1,720	,910	3,574	1	,059	,179
Coag. disorder (Quick,%)	-,024	,015	2,649	1	,104	,977
Preop. Bilirubin (mg/dL)	-,010	,049	,042	1	,837	,990
Preop. GOT (U/L)	,005	,012	,155	1	,693	1,005
Preeop. GPT (U/L)	-,001	,005	,052	1	,820	,999
Preop. AP (U/L)	,002	,004	,239	1	,625	1,002
Preop. Leucocytes (/nL)	-,158	,139	1,288	1	,256	,854
Preop. CRP (mg/dL)	,001	,029	,002	1	,966	1,001
OP-Duration (min)	,008	,004	4,166	1	,041	1,008

### Secondary endpoints

**Fig. 2:**
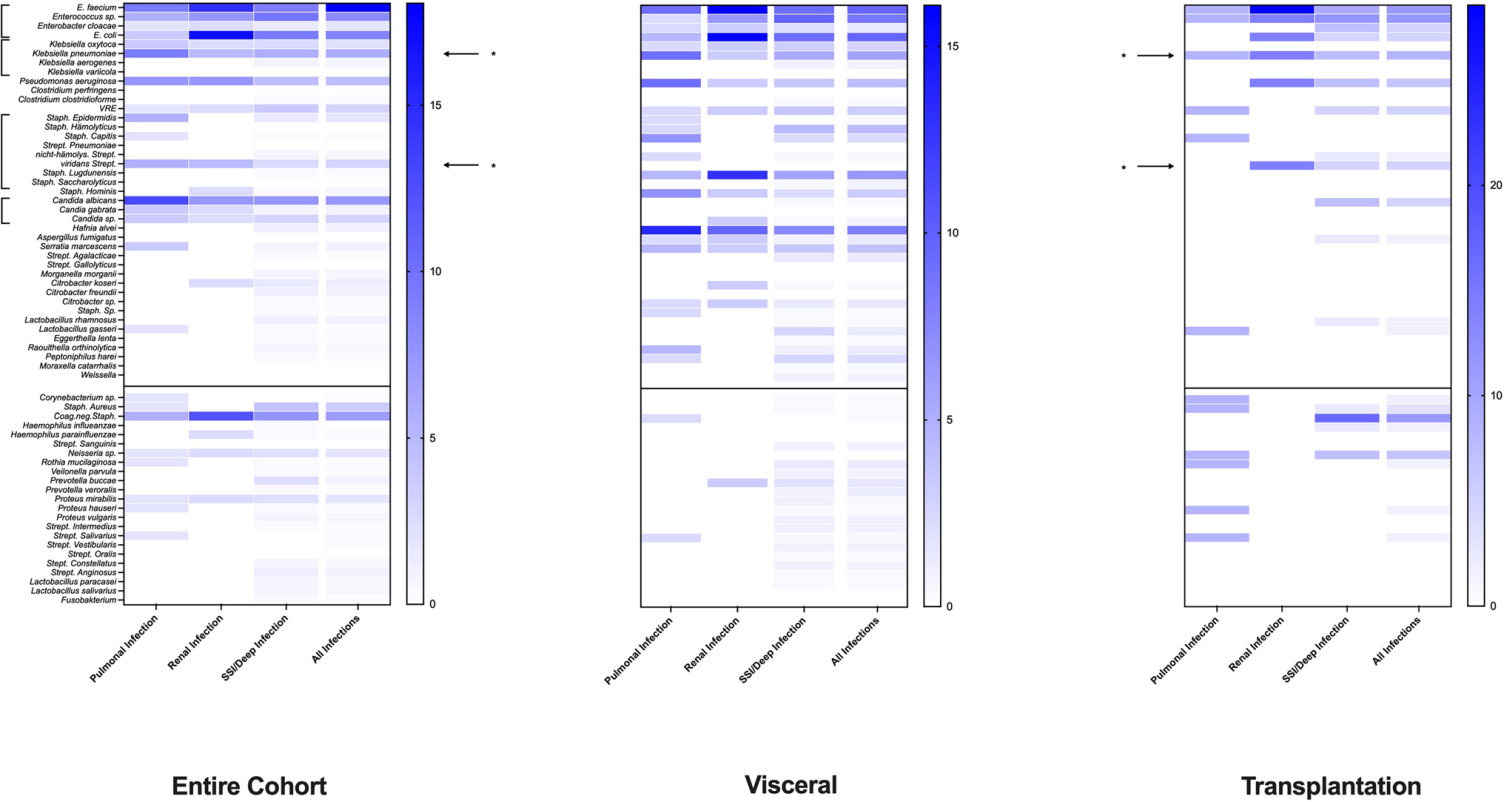
Heat map of the distribution of germs in smears from patients with infectious complications (the transplant cohort is compared here with the overall cohort and the visceral surgery cohort). The blue color intensity codes the frequency (%) with which each pathogen was detected in the swabs of a specific complication group (pulmonary vs. renal vs. SSI); oral pathogens are listed under the horizontal black line. * indicate the oropathogenic germs Klebsiella pneumoniae and viridans streptococci, which are associated with diseases such as IBD, CRC or esophageal carcinoma

In the entire cohort, ten patients (2.4%) died within the first 30 days postoperatively. With the exception of the preoperative elevated GOT value (*p* = 0.007), no other factors significantly influenced the 30-day mortality rate. The average length of hospital stay was 19.43 days (SD = 17.35). Preoperative steroid use (*p* = 0.013), preoperative ascites (*p* = 0.035), bleeding tendency (*p* = 0.014) and duration of surgery (*p* = 0.017) were identified as significant influencing parameters for the overall stay. The average duration of intensive care was 5.52 days (SD = 9.70). Significant parameters influencing the duration of intensive care were the preoperative bilirubin value (*p* = 0.016) and the duration of surgery (*p* = 0.021). A microbe normally belonging to the commensal oral bacterial flora was identified as a source of infection in 67 (55.4%) patients with infectious complications and in 42 (34.7%) poor dental status. The occurrence of these microbes in bacteriological analyses from patients with infection-related complications is shown in Fig. 2 shown. Using Spearman correlation, a significant relationship between oral microbes and infection-related postoperative complications rates was observed (*p* < 0.001). In support of this, a significant correlation between preoperative poor dental status and oral microbes present in postoperative bacteriological examinations could be demonstrated (*p* = 0.045). In 35 (8.4%) patients with preoperative poor dental status, microbes typically belonging to the oro-phaynx bacterial flora were found postoperatively in the surgical area.

In the visceral surgery cohort, 10 patients (3.3%) died within 30 days following surgery. The preoperative GOT value was identified as the only significant factor influencing 30-day mortality (*p* = 0.008). 166 patients (54.2%) had a total hospital stay of more than 15 days. The average length of hospital stay was 21.55 days (SD = 18.53). The GPT value was identified as the only significant factor influencing the length of hospital stay (*p* = 0.042). In this subgroup, 25 patients (8.2%) had an intensive care stay of more than 15 days and the average duration of the intensive care stay was 6.48 days (SD = 11.08). The preoperative bilirubin value (*p* = 0.018) and the duration of surgery (*p* = 0.028) proved to be significant influencing factors with regard to the intensive care stay. An analysis of microbes in patients with infection-related complications showed 97 (31.7%) patients with infectious complications and an existing bacterial spectrum. In 60 (19.6%) cases, there was positive bacterial detection, but no infectious, postoperative complications (as some patients did undergo routinely swabs). Oral microbes were detected in 28 (9.2%) patients with a poor dental status. Overall, a significant influence of oral microbes on postoperative complications was shown in this subgroup using the Spearman correlation (*p* < 0.001). However, no significant correlation between oral bacteria and poor dental status could be demonstrated (*p* = 0.156).

In the transplant surgery cohort, there were no deaths within the first 30 postoperative days. The average length of hospital stay was 13. 61 days (SD = 11.85). Dental status (*p* = 0.040), preoperative steroid use (*p* = 0.018), bleeding tendency (*p* =  < 0.001), preoperative bilirubin (*p* = 0.010), and GOT (*p* = 0.005) were identified as significant factors influencing the length of hospital stay. The average duration of intensive care was 2.86 days (SD = 2.36). An interpretation of the logistic regression analysis could not be performed due to a too large standard error. The microbial spectrum of patients who suffered an infection-related complication is shown in Fig. 2. It is noticeable here that the distribution of microbes in the transplant group differs significantly from the overall and visceral surgery cohorts. However, the analysis shows, as in the overall cohort, that the oropathogenic germs Klebsiella pneumoniae and Streptococci viridans are involved in a significant number of postoperative infectious complications. In 7 (6.3%) patients with preoperative poor dental status, an oral bacterial was detected in the surgical area. In this subgroup, the Spearman correlation also demonstrated a significant association between oral bacteria and infection-related complications (*p* = 0.017). No significant association between dental status and oral bacteria could be shown (*p* = 0.132).

## Discussion

In the present series, age (*p* = 0.049), BMI (*p* = 0.035) and quick value (i.e. a surrogate maker for synthetic liver function) (*p* = 0.033) were all associated with the occurrence of postoperative complications. This result confirms previously published studies, which demonstrated advanced age (> 80 years) [43–45], obesity or increased BMI [46], preoperative abdominal surgery [45], hypertension or presence of coronary disease [47], coagulation disorders, hepatic impairment, preoperative steroid use [6, 37, 48–55], the presence of preoperative renal insufficiency [56] and the duration of surgery [57] to be significant risk factors for postoperative morbidity in surgical series.

In the present investigation, infection-related complications occurred in 29% of patients and accounted for approximately 45% of total complications. This incidence following major abdominal procedures, regardless of the type of procedure (visceral or transplantation surgery), is comparable to previous published series (11% to 30%) [58–61]. Risk factors for the development of an infection-related complication in this population were dental status (*p* = 0. 034) and duration of surgery (*p* = 0.027). The association between dental status and postoperative infectious complications has already been established after vascular-[25, 62–64], cardiac- [2, 4, 5, 7, 9, 11, 30, 65–67] and orthopaedic surgery [3, 26, 29, 68].

It is therefore important that patients scheduled for these elective surgeries are thoroughly assessed for the presence oro-pharyngeal infections and that these are adequately treated prior to surgery. Such an association has not yet been clearly demonstrated in the context of major abdominal surgery, with the exception of a few studies [1, 6, 7], The current work is therefore of particular importance, as it seems to demonstrate that dental status also plays an important role in the development of postoperative infection-related complications not only in patients undergoing organ transplantation, but also in patients undergoing major visceral surgery (e. g. esophageal resection, pancreatic resection, liver resection). Dental status (*p* = 0.043), preoperative ascites (*p* = 0.006), bleeding tendency (*p* = 0.021), bilirubin (*p* = 0.008) and GPT (*p* = 0.041) were shown to associated with postoperative infection-related complications.

As with liver function [69–74], dental status has been linked to the incidence of postoperative infection-related complications [2–5, 25, 26, 29, 62, 63, 67]. In order to determine the magnitude of this association, in addition to examining causality, prospective studies are required. Moreover, such studies could also examine the extent to which routine preoperative dental assessment and treatment could acutally reduce the overall incidence of postoperative infection-related complications. If demonstated, such an approach may reduce postoperative morbidity and mortality as well as reducing health care costs and could be incorporated into routine surgical guidelines.

In the transplantation subgroup, logistic regression analysis showed that only the duration of the operation (*p* = 0.041) was associated with the incidence of infectious complications. In this group, the association between oral microbes and postoperative infectious complications could be demonstrated (*p* < 0.001). In this subgroup, dental status did not appear to be significantly associated with postoperative infectious complications. Transplanted patients undergo preoperative screening in order to overcome possible complications linked with immunosuppression after transplantation, as recommended by existing evidence [27, 41, 42, 75]. Intensive immunosuppression is required after each organ transplant to prevent rejection [39]. However, this immunosuppression may increase vulnerability to oral-bacteria related infection and disease [38, 39]. Foci of infection may adversely affect the long-term prognosis of the transplanted organ if they are not adequately addressed preoperatively. There is evidence that postoperative invasive dental surgery may promote graft rejection due to the associated bacteremia [38, 40]. Poor oral status also appears to have a negative impact on postoperative graft infections [41, 76]. Therefore, in several studies, in spite of low-grade evidence before transplantation, preoperative rehabilitation with antibiotic prophylaxis is recommended [27, 38, 40, 75–77]. This may well have explained the lack of an association between oral bacteria and postoperative infection-related complications in our cohort.

Pathogenic bacteria, which are particularly associated with dental diseases, also occur in part in the current microbial analysis and in this respect confirm previous studies. Examples of oropathogenic bacteria are *Streptococcus viridans* or *Klebsiellae*. These microorganisms seem to be linked to the pathomechanism of diseases including inflammatory bowel disease, rheumatoid arthritis, non-alcoholic steatohepatitis (NASH) or even in the development of colorectal carcinoma (CRC) or esophageal carcinoma [21, 36, 79–83]. The importance of bacteria of dental origin in the development of these complications therefore seems to be confirmed by the spectrum analysis. This analysis also highlights the importance of preventive treatment for this source of infectious complications. The bacterial samples spectrum analysis (Fig. 2) could prove that: (1) SSI/Deep Space infections are mostly caused by a mixed flora in which both skin, intestinal and oropharyngeal germs are present, (2) pulmonary infectious complications are more likely to be caused by oropharyngeal and intestinal germs, and (3) renal infectious complications are caused by oropharyngeal and intestinal germs. Thus, there appears to be an important association between oral germs and pulmonary, renal, and SSI complications. Numerous studies to date have demonstrated oral cavity microorganisms in various surgical sites [3, 5, 12, 26, 78].

As far as the secondary endpoints of our study are concerned, dental status appears to be only associated with length of hospital stay in transplant patients. A connection with the 30-day mortality rate and the duration of intensive care treatment could not be proven, neither for the entire or visceral subgroup, nore for the transplantation subgroup.

### Limitations

The study may have been subject to selection bias based on the low number of patients in each subgroup, its retrospective nature and the monocentric setting. With regard to the investigation of the influence of dental status, this limitation is reinforced by the fact that in the transplantation group the dental status is routinely examining and documented preoperatively. Prospective studies are required to confirm the associations we have reported and to shed light on causality. Moreover, an extended period of follow-up (i.e. longer than the standard 30 day cut-off) would be desirable given that complications in transplant patients tend to occur later than those in patients who have undergone visceral surger [38]. An important limitation of the current study is that the dental status was not systematically assessed by dentists. In most cases, the assessment of the dental status is limited to that by anaesthetists as part of the preoperative assessment. However, this does reflect routine clinical “real-world” practice. As the complications were retrospectively assessed, grade I and II complications according to Clavien-Dindo classification might be unreliably assessed. The microbial analysis was only performed in a small percentage of patients with infection-related postoperative complications. This undoubtedly impacted upon the analysis, compounded by the small number of patients in each subgroup. In addition, prospective studies would be well-advised to examine the oral microbial flora in all patients prior to surgery, ideally using 16S-based next generation sequencing to avoid the bias associated with bacterial culture [84].

## Conclusions

We report a significant correlation between preoperatively assessed dental status and incidence of infection-related postoperative complications in patients who underwent visceral surgery. Furthermore, through a more detailed analysis of postoperative microbial samples a significant correlation was found between oral microorganisms and postoperative complications. This was not the case in the transplantation subgroup. It is increasing recognised that the oral and gastrointestinal microbiome influences overall health. However, less well appreciated the extent to which these resident bacterial populations impact upon the incidence of postoperative infection-related complications. If our findings can be replicated in prospective studies, the case for routine preoperative oral and dental assessment for all patients undergoing elective visceral surgery may become persuasive and result in significant changes in preoperative assessment and standards.

## Data Availability

The data that support the findings of this study are not openly available due to reasons of sensitivity and are available from the corresponding author upon reasonable request.

## Abbreviations

*AP*: Alkaline Phosphatase
*BMI*: Body Mass Index
*CCI*: Charlson Comorbidity Index
*CRC*: ColoRectal Carcinoma
*CRP*: C-Reactive Protein
*GOT*: Aspartate Aminotransferase
*GPT*: Alanine Aminotransferase
*IBD*: Inflammatory Bowel Disease
*ICU*: Intensive Care Unit
*IMC*: Intermediate Care
*LOS*: Length of Stay
*NASH*: Non-Alcoholic SteatoHepatitis
*SD*: Standard Deviation
*SOT*: Solid Organ Transplantation
*SSI*: Surgical Site Infection
*WHO*: World Health Organization
